# Tumor cell-derived asymmetric dimethylarginine regulates macrophage functions and polarization

**DOI:** 10.1186/s12935-022-02769-7

**Published:** 2022-11-15

**Authors:** Yi-Ling Chen, AKaychia T. Lowery, Samuel Lin, Ameae M. Walker, Kuan-Hui E. Chen

**Affiliations:** 1 Department of Electronic Engineering, National Kaohsiung University of Science and Technology, Kaohsiung, Taiwan; 2 Division of Mathematics and Sciences, Delta State University, 38733 Cleveland, MS USA; 3 Division of Biomedical Sciences, School of Medicine, University of California Riverside, 92521 Riverside, CA USA; 4 Department of Biological Sciences, Texas Tech University, 79409 Lubbock, TX USA

**Keywords:** Arginine metabolism, Asymmetric dimethylarginine, cancer stem cells, Autophagy, Macrophage polarization

## Abstract

**Background:**

Asymmetric dimethylarginine (ADMA), which is significantly elevated in the plasma of cancer patients, is formed via intracellular recycling of methylated proteins and serves as a precursor for resynthesis of arginine. However, the cause of ADMA elevation in cancers and its impact on the regulation of tumor immunity is not known.

**Methods:**

Three mouse breast cell lines (normal breast epithelial HC11, breast cancer EMT6 and triple negative breast cancer 4T1) and their equivalent 3D stem cell culture were used to analyze the secretion of ADMA using ELISA and their responses to ADMA. Bone marrow-derived macrophages and/or RAW264.7 cells were used to determine the impact of increased extracellular ADMA on macrophage-tumor interactions. Gene/protein expression was analyzed through RNAseq, qPCR and flow cytometry. Protein functional analyses were conducted via fluorescent imaging (arginine uptake, tumor phagocytosis) and enzymatic assay (arginase activity). Cell viability was measured via MTS assay and/or direct cell counting using Countess III FL system.

**Results:**

For macrophages, ADMA impaired proliferation and phagocytosis of tumor cells, and even caused death in cultures incubated without arginine. ADMA also led to an unusual macrophage phenotype, with increased expression of arginase, cd163 and cd206 but decreased expression of il10 and dectin-1. In contrast to the severely negative impacts on macrophages, ADMA had relatively minor effects on proliferation and survival of mouse normal epithelial HC11 cells, mouse breast cancer EMT6 and 4T1 cells, but there was increased expression of the mesenchymal markers, vimentin and snail2, and decreased expression of the epithelial marker, mucin-1 in EMT6 cells. When tumor cells were co-cultured ex vivo with tumor antigen in vivo-primed splenocytes, the tumor cells secreted more ADMA and there were alterations in the tumor cell arginine metabolic landscape, including increased expression of genes involved in arginine uptake, metabolism and methylation, and decreased expression of a gene that is responsible for arginine demethylation. Additionally, interferon-gamma, a cytokine involved in immune challenge, increased secretion of ADMA in tumor cells, a process attenuated by an autophagy inhibitor.

**Conclusion:**

Our results suggest initial immune attack promotes autophagy in tumor cells, which then secrete ADMA to manipulate macrophage polarization favoring tumor tolerance.

**Supplementary Information:**

The online version contains supplementary material available at 10.1186/s12935-022-02769-7.

## Background

A tumor is a heterogeneous mass of cells, containing parenchymal cancer cells, supportive cells (ex, fibroblasts, pericytes and adipocytes) and tumor-infiltrating cells, many of which are immune cells [1]. The presence of tumor infiltrating immune cells is of significance due to their potential contributions to tumor clearance or tolerance [2, 3]. Among the tumor infiltrating immune cells, T cells are the most potent anti-cancer immune cells [4]. Low infiltration of T cells and high inhibition of their functions constitute the major barriers to anti-tumor immunity [5]. In contrast to the T cells, macrophages are the most abundant cells, accounting for ~ 30–50% of the infiltrating immune cells within the tumor microenvironment [6, 7]. Most tumor macrophages are recruited from circulating monocytes, themselves produced in the bone marrow [8, 9]. The remaining tumor macrophages may be self-renewing local resident macrophages, originally derived from the yolk sac, or other recruited macrophages from additional resident sources such as the liver or spleen [10, 11]. Given their abundance in a tumor, functional manipulation of tumor macrophages has the potential to determine the outcome of tumor progression.

Part of the innate immune system, macrophages perform diverse immune functions, including directly phagocytosing pathogens and dying cells and inciting T cells to mount an immune attack [12]. However, in several human diseases like cancer, macrophages display plasticity, rendering them either pro- or anti-tumor. This plasticity allows the macrophages to develop along a spectrum of subtypes between those known as M1 and M2. At one extreme, the M1 subtype macrophages kill pathogens, engulf tumor cells, and present antigens to activate T cells [13]. At the other extreme, the M2 macrophages secrete factors that promote tumor growth, angiogenesis, and metastasis [14, 15]. The amino acid, arginine, plays a central role in the management of the M1/M2 macrophage dichotomy. Arginine metabolism in macrophages can drive the generation of M1 anti-tumor macrophages if metabolized by nitric oxide synthase (NOS) or the generation of M2 pro-tumor macrophages if metabolized by arginase (ARG) [16]. A better understanding of arginine metabolic pathways in macrophages may lead to strategies promoting a switch from M2 to M1 in tumor macrophages, thereby increasing anti-tumor immunity.

As a non-essential amino acid, arginine can be either acquired from the environment or synthesized by cells. Multiple routes of arginine production have been described, including the urea cycle [17], a shunt off the citric acid cycle from L-aspartate [17–19], and proline and glutamine metabolism [20, 21]. Another possible route, though less discussed, is proteolysis. Proteolysis of post-translationally methylated proteins yields asymmetric dimethylarginine (ADMA) [22]. Once produced, the majority of ADMA is degraded by dimethylarginine dimethylaminohydrolase (DDAH) into citrulline, which can then be recycled and used for arginine synthesis [22]. Only a small proportion of ADMA is normally released into the circulation and excreted in urine. Interestingly, ADMA inhibits NOS activity, preventing nitric oxide production in macrophages in many animal models of disease [23–25]. However, unknown is whether inhibition of NOS by ADMA encourages the production of M2 macrophages. Also unknown is whether tumor cells secrete ADMA, thereby switching tumor macrophages into the M2 subtype.

Elevated levels of ADMA are seen in patients with hypertension, chronic heart disease, diabetes mellitus and chronic renal failure because of ADMA’s inhibitory effect on NOS of endothelial cells, thereby preventing the generation of nitric oxide and the dilation of blood vessels [26]. Although its importance in the regulation of cardiovascular function is well known, little is understood about ADMA in tumors. Current knowledge includes the elevation of serum ADMA in both human cancer patients and the mouse breast cancer 4T1 model [27–30]. Most other publications have focused on the role of ADMA in tumor angiogenesis/tumor cell migration and, even in that context, there is some controversy. For example, in gastric cancers, increased serum ADMA is associated with tumor metastasis [31], whereas increased ADMA in prostate cancer is associated with reduced tumor angiogenesis and metastasis [32]. This discrepancy in outcome may be the result of effects of ADMA on tumor immune cells. While ADMA production may impact tumor angiogenesis through effects on endothelial cells, it may also protect the tumor through the restrained infiltration of effector T cells. The impact of ADMA on immune cells has never been addressed and its determination could provide more insights for tumor regulation.

Cancer deaths primarily occur because of metastatic spread of tumor cells to crucial organs. However, not all tumor cells disseminated from the primary tumor can grow into metastases. A subpopulation of cancer cells, known as cancer stem cells (CSCs), which exhibit stem cell properties such as self-renewal and asymmetric division, have enhanced invasion and migratory capabilities, making them important in metastasis [33]. While ADMA elevation is seen in cancer, the source of this ADMA in patients has not been identified. Whether parenchymal tumor cells or CSCs produce ADMA is also unknown.

Here, we have shown ADMA impacts macrophages through temporary inhibition of tumor phagocytosis, reduced proliferation, reduced survival in response to a nutrient shortage, and polarization towards an unusual M2-like phenotype. Additionally, we demonstrate that both bulk tumor cells and CSCs contribute to the production of ADMA. Furthermore, autophagy, potentially initiated by interferon-γ, serves as a mechanism for the generation of ADMA by tumor cells. Thus, our work depicts a new regulatory role for ADMA in tumor-immune interactions.

## Methods

### Tumor cell culture and tumor stem cell enrichment

Mouse breast cancer 4T1 cells (CRL-2539™, ATCC), EMT6 (CRL-2755™, ATCC), and normal mouse mammary HC11 cells (CRL-3062™, ATCC) were routinely cultured in RPMI 1640 (10-040-CV, Corning) with 10% FBS (10082-147, Gibco) and 1% penicillin plus streptomycin supplement (SV30010, Citiva) at 37 °C in a high humidity, 5% CO2 and 95% air incubator. Three-dimensional cancer stem cell (CSC) spheroid culture was performed as previously described [34, 35]. Briefly, 4T1 cells were cultured using ultralow attachment plates (3471, Corning) in serum free RPMI1640 supplemented with 2% SM1 supplement (5711, StemCell Technologies), 20 ng/mL EGF (78,006, StemCell Technologies), 20 ng/ml FGF (78,003, StemCell Technologies) and 4 µg/ml heparin (7980, StemCell Technologies) for 7 days. Under these conditions, tumor cells with stemness continue to proliferate and grow into spheres, while other cells in the culture are either unable to proliferate or die. At day 7, CSC spheroids, greater than 40 μm, were enriched and collected using a 40 μm cell strainer (50-196-0596, ThermoFisher). CSC spheroids smaller than 40 μm and non-proliferating non-CSCs pass through the strainer and are discarded.

### RNAseq of tumor stem cells in response to an active immune environment

Eight-week-old female Balb/c mice were injected with syngeneic 4T1 tumor cells into the mammary fat pad and housed for 28 days. At day 28, spleens were collected and gently mashed through a cell strainer with 5 mL serum-free RPMI 1640. Red blood cells in this cell suspension were removed using ACK lysis buffer (A1049201, Gibco). Cell suspensions were then pelleted as splenocytes. These in vivo tumor-activated splenocytes were then either incubated in Dulbecco’s phosphate buffered saline (DPBS) or were reinforcedly activated using anti-CD3/CD28 dynabeads (11452D, Gibco) in the presence of IL2 (I2644, Sigma). These splenocytes (tumor primed or tumor primed plus reinforced activation) were pipetted to generate an even single cell suspension and then co-cultured with CSC spheroids (CSCsp) in the same 3D culture condition mentioned above.

The CSCsp were isolated from the ex vivo co-culture with splenocytes using a 40 μm cell strainer and washed multiple times with DPBS until all single splenocytes were filtered and CSCsp were retained in the cell strainer. RNA from CSCsp was extracted using Trizol (15,596,018, Invitrogen). The same amount of RNA (500 ng/each) from 4 independent experiments was multiplexed for RNAseq (Novogene, CA). The RNAseq results were analyzed using the Hisat2 (v2.2.0)/Stringtie (v2.1.3) pipeline and data are available on NCBI Gene Expression Omnibus (GEO) with the accession number GSE199983.

### Macrophage culture and Ex vivo bone marrow derived macrophage culture

Mouse macrophage RAW264.7 cells (91,062,702, Sigma) were routinely cultured in RPMI 1640 with 10% FBS and 1% pen/strep supplement at 37 °C in a humidified 5% CO_2_ and 95% air incubator. Mouse fibroblast L929 cells (85,011,425, Sigma) were cultured under the same conditions and the conditioned medium derived from the L929 cell culture was diluted and used as a source of macrophage colony stimulating factor for bone marrow stem cell differentiation into macrophages [36]. For the bone marrow-derived macrophages, bone marrow was harvested from five-month-old Balb/c mice (000651, Jackson Laboratory) and cultured in RPMI 1640 with 10% FBS, 1% pen/strep supplement and 30% L929-derived conditioned medium at 37 °C in a humidified 5% CO_2_ and 95% air incubator for 7 days. Production of bone marrow-derived macrophages was confirmed at day 7 by the expression of F4/80 using flow cytometry, and 97% of the cells were validated as differentiated F4/80^+^ macrophages. The RAW264.7 cells were established from the genetic strain Balb/c mice (the same background as our bone marrow derived macrophages).

### Phagocytic analyses of macrophages

5 × 10^5^ bone marrow-derived macrophages or RAW264.7 cells were seeded in 6 well plates and treated with either DPBS or ADMA (5 mM in DPBS) (D4268, Sigma) for 3 days. At day 3, 5 × 10^5^ of CFDA-SE (V12883, ThermoFisher)-stained 4T1 cells were added to the culture. At 10 min intervals, the medium was agitated by gentle pipetting to resuspend the fluorescent 4T1 cells to prevent their attachment to the plate and therefore loss from the medium. A control group with fluorescent 4T1 cells only in the plate was used as a determinant of potential attachment of 4T1 cells during the protocol. At the end of the phagocytosis incubation, any cells that may have attached to the plate were trypsinized and analyzed for fluorescence to control for any loss of fluorescence in the media as a result of attachment despite the pipetting and to count the exact macrophage numbers after the 3-day pretreatment with DPBS or ADMA. The number of fluorescent 4T1 cells remaining in the medium was analyzed using a Countess III FL counter (A49866, ThermoFisher) as the non-phagocytosed tumor cells. The phagocytic rate per macrophage was calculated as “total number of consumed fluorescently labelled 4T1 cells divided by the number of macrophages on the plate”. ADMA is taken into cells through Slc7a2, a transporter for cationic amino acids such as arginine, lysine, ornithine, etc. The concentrations of arginine and Lysine in RPMI 1640 are 1.16 mM and 0.27 mM, respectively. Thus, arginine, lysine and ornithine are natural competitors for ADMA uptake. Additionally, different isoforms of Slc7a2 show differential preferences to ADMA and l-arginine uptake. The Slc7a2A has about a 2-fold greater efficiency in transporting l-arginine than ADMA, while the Slc7a2B does not have such preference [42]. Furthermore, it has been shown that ADMA fails to compete for arginine uptake unless mM concentrations are used [37]. The ADMA concentration for treatment was therefore set at 5 mM  (1 mg/mL)  to ensure the uptake of ADMA into cells.

### Growth analyses of macrophages and tumor cells by ADMA treatment

The growth analyses of macrophage were examined by direct cell counting using a Countess III FL counter. The macrophage cell line, RAW264.7, was cultured in complete RPMI1640 and treated with either DPBS or ADMA (5 mM in DPBS) for 3 days. The number of macrophages was normalized to the initial seeding number (5 × 10^5^) to determine the impact of ADMA on macrophage proliferation or death. Since arginine and lysine in the culture medium serve as competitors for ADMA uptake, the absolute impact of ADMA was also assessed by culture in arginine- and lysine-free medium.

For the growth/survival analyses of normal/tumor cells in response to ADMA treatment, 3 × 10^4^ of mouse breast cells (mouse normal-like HC11, breast cancer EMT6, and TNBC 4T1) were seeded in 96 well plate and treated with DPBS or ADMA (5 mM) in complete RPMI 1640 or Arginine/Lysine-free RPMI 1640 for 3 days. At day 3, MTS cell proliferation assay was performed following the manufacturer’s instructions.

### Arginine metabolic analyses in tumor cells

To explore arginine uptake by CSCs in response to different extents of immune threats, CSCsp not exposed to splenocytes, exposed to in vivo-activated splenocytes or exposed to in vivo and anti-CD3/CD28 Dynabeads activated splenocytes were cultured in the Arginine/Lysine-free RPMI 1640 with 20 nM Dansyl-Arginine (D0250, Sigma) for 30 min. The resultant green fluorescence in the CSCsp was examined by fluorescence microscopy. To investigate if activities of enzymes involved in arginine metabolism increased in tumor cells after experiencing immune interactions, the enzymatic activity of arginase (ARG) was conducted through the measurement of urea using an Arg activity assay kit (MAK112, Sigma). To determine whether increased expression of *prmt1* and decreased expression of *jmjd6* led to the production and secretion of ADMA by tumor and tumor stem cells, we performed an ADMA ELISA (MBS2100173, MyBioSource) of the conditioned medium.

### Autophagy analysis

To determine if autophagy served as a major manner for ADMA production by tumor cells, we first examined if the cytokine IFN-γ, released from effector T cells, initiated tumor cell autophagy using the Premo Autophagy Sensor LC3B RFP kit (P36236, ThermoFisher). Briefly, 5 × 10^5^ mouse 4T1 breast cancer cells were seeded in 35 mm dishes and treated with DPBS (control), 0.2 ng/mL IFN-γ (low dose known not to induce autophagy), and 200 ng/mL IFN-γ (high dose known to induce autophagy in hepatocellular tumor cells) for 2 days. At the end of day 2, cells from each treatment were transduced with Premo Autophagy Sensor LC3B RFP (a multiplicity of infection of 30 plaque forming units was used) and cultured for another 24 h. The resultant red fluorescence, which indicates autophagy, was examined by fluorescence microscopy.

To investigate if the IFN-γ directed autophagy impacted ADMA production, 3 × 10^4^ mouse mammary cells (mouse HC11, EMT6, 4T1) were seeded in 96 well plates and treated with DPBS, 200 ng/mL IFN-γ, or 200 ng/mL IFN-γ together with 250 nM autophagy inhibitor (5.34360, Sigma) for 3 days. Conditioned medium was then collected and assayed for ADMA by ELISA.

### qRT–PCR

To explore the impact of ADMA on macrophage cells (RAW264.7) or tumor cells (mouse breast cancer EMT6), expression of appropriate genes was analyzed by real-time PCR. Briefly, 5 × 10^5^ cells (RAW264.7 or EMT6) were seeded in 6 well plates and treated with DPBS or 5 mM ADMA for 3 days. Cells were then harvested, RNA was extracted using Trizol, and cDNA synthesis was conducted using a RevertAid RT Reverse Transcription Kit (K1691, ThermoFisher). Real-time PCR was conducted on a Chai open qPCR system (Chai, CA). Primers used can be found in Additional file 2: Table S2. Gene expression analyses were normalized to the housekeeping control, β-actin.

### Flow cytometry analysis

Macrophages were treated with DPBS or 1 mg/mL ADMA for 3 days and then subjected to flow cytometric analysis of surface expression of two M2 markers, CD163 and CD206. Cells were first Fc-blocked and stained with fluorophore-conjugated antibodies for surface CD163 (12-1631-82, ThermoFisher) and CD206 (MA5-16870, ThermoFisher). All flow cytometry work was performed on a BD FACS Canto II and data were analyzed using Flowjo software.

### Statistical analysis

All results are presented as mean ± SD. Statistical analysis was generally performed by paired t-test for comparison between two groups. For comparisons of more than two groups and data from in vitro studies, significance was determined by ANOVA using posttests (Tukey’s HSD for equal sample size and Scheffe for unequal sample size) and Bonferroni correction for multiple comparisons against a single control group. Two tailed analysis was used. Results were considered significant when p ≤ 0.05. All graphs were generated using GraphPad Prism software (version 8.01).

## Results

### Modulation of macrophage functions by ADMA

**Fig. 1:**
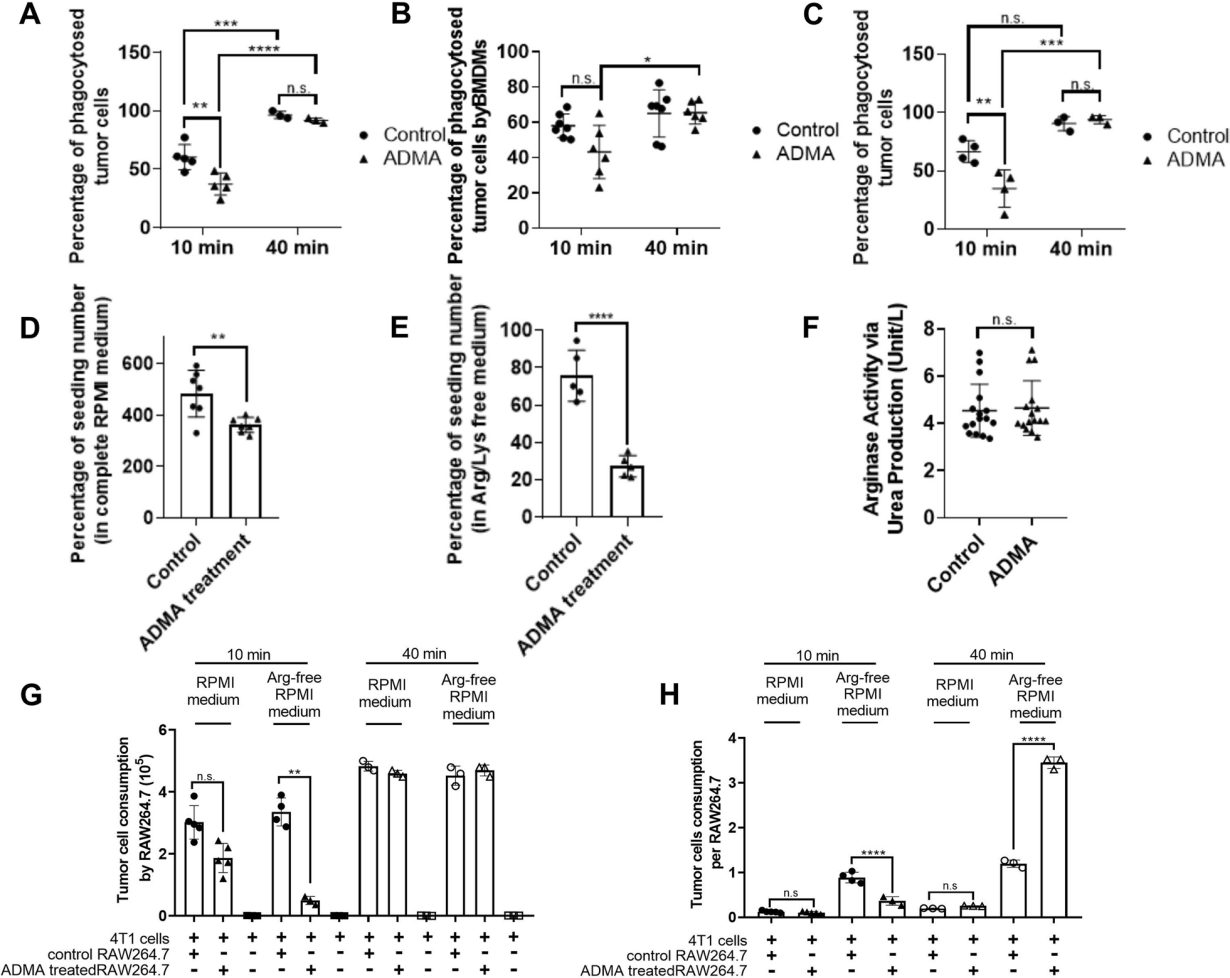
Effects of ADMA on macrophage number and phagocytic ability. Phagocytosis of fluorescent tumor cells after a 3-day prior ADMA (5 mM) treatment in regular RPMI 1640 medium by RAW264.7 cells (**A**) or bone marrow-derived macrophages (BMDMs) (**B**). Phagocytosis of fluorescent tumor cells after a 3-day prior ADMA (1 mg/mL) treatment in arginine- and lysine-free RPMI by RAW264.7 cells (**C**). Effect of the 3-day incubation in ADMA on RAW264.7 cell number in regular RPMI 1640 medium (**D**) and arginine- and lysine-free RPMI 1640 medium (**E**). The 3-day ADMA treatment (1 mg/mL) did not enhance arginase activity in RAW264.7 cells, as measured by the production of urea collected in the conditioned medium (**F**). As ADMA impacts the proliferation (**D**) and survival (**E**) of RAW264.7 cells, fluorescent 4T1 cell consumption was further presented as cell number overall (G) and per RAW264.7 cell (H). Data are presented as individual replicates with the mean ± SD. (*p < 0.05, ** p < 0.01, ****p < 0.0001), N = 3–5 (**A**, **B**); N = 6–7 (**C**); N = 7 (**D**); N = 5 (**E**); N = 16 (**F**); N = 3–5 (**G**, **H**).

As the majority of immune cells infiltrating most tumors are macrophages and arginine plays important roles in macrophage functions, we sought to determine the impact of ADMA on macrophages, first by using the syngeneic RAW264.7 cell line. RAW264.7 cells were cultured for 3 days with ADMA (1 mg/mL in DPBS) or diluent as control. At day 3, fluorescently CFDA-SE-stained mouse 4T1 bulk breast cancer cells were added to the culture for 10 or 40 min. The medium containing non-phagocytosed residual fluorescent 4T1 cells was then separated from the adherent RAW264.7 cells to determine the relative tumor cell phagocytic function of the macrophages by subtraction from the number of fluorescent 4T1 cells of the starting suspension. The group with only 4T1 cells in Fig. 1 (Fig. 1G) was used to validate the loss of fluorescent 4T1 cells in the media as a measure of phagocytosis of tumor cells by determining that loss from the media was not the result of 4T1 cell attachment.

At 10 min after fluorescent 4T1 cells were added to the culture, only 44% of 4T1 cells were taken up in the ADMA treated group compared to 60% uptake in the control group. However, at 40 min, almost all fluorescent tumor cells were phagocytosed by RAW264.7 cells in both groups (95% tumor cell taken in control and 91% in ADMA) (Fig. 1A).

As most tumor macrophages are derived from the bone marrow and recruited from the circulation, we sought to validate the physiological significance of the ADMA effect by using bone marrow-derived macrophages. Bone marrow cells were collected from 5-month old Balb/c mice and stimulated to differentiate into macrophages using L929 cell-derived conditioned medium, as described previously [36]. The bone marrow derived macrophages were then treated with ADMA (1 mg/mL) or vehicle control for 3 days. At day 3, fluorescent 4T1 cells were added to the culture to initiate tumor cell phagocytosis. Similar to what was seen for the RAW264.7 cells, the ADMA treated bone marrow-derived macrophages showed a lower phagocytic rate at the 10 min time point (43.1%) compared to the control bone marrow-derived macrophages (57.9%), but because of greater variation in these cultures, the difference was not statistically significant (Fig. 1B). Longer exposure of bone marrow-derived macrophages did not increase fluorescent 4T1 tumor cell phagocytosis, with no change between the short (10 min, 57.9% of phagocytotic rate) and long (40 min, 64.9% of phagocytotic rate) co-culture in the control group (Fig. 1B) This implies that some additional development or stimulus is required to establish the complete phagocytic capacity seen in the RAW264.7 cells. However, there was an increase in tumor phagocytosis with increased co-incubation in the ADMA treated bone marrow-derived macrophages (43.1% at 10 min and 65.3% at 40 min) (Fig. 1B). Again, these bone marrow derived macrophages were unable to engulf all 4T1 cells as efficiently as the RAW264.7 cells at the 40 min time point but a catch-up phenomenon of tumor phagocytosis was observed for the ADMA-treated bone marrow-derived macrophages at 40 min. Overall, work with the primary cells was consistent with that found in RAW264.7 cells in-as-much as ADMA treatment appeared to delay tumor cell phagocytosis and, at 40 min, the tumor phagocytosis rate was the same between control and ADMA treated.

In culture medium, arginine and lysine serve as competitors for ADMA uptake. We therefore conducted the experiment again using arginine- and lysine-free medium. In such medium, the apparent delay in phagocytosis became more pronounced (61% of tumor cells removed in the control group versus 34% in ADMA group at 10 min) (Fig. 1C). Again, there was no difference at the later time point (92% tumor cell taken in control group and 96% in ADMA group) (Fig. 1C).

We next determined the impact of ADMA on macrophage (RAW264.7) cell number. As seen in Fig. 1D, ADMA treatment reduced the proliferation rate during the 3-day period by 25% (4.8 fold seeding number in the control versus 3.6 fold in the ADMA treated group) (Fig. 1D). In arginine- and lysine-free medium, not only did the cells not proliferate but they also died (75% survival in the control versus 27% survival in the ADMA treated group) (Fig. 1E). The greater death rate of macrophages in ADMA in arginine- and lysine-free medium implied an inability of macrophages to convert ADMA to citrulline for arginine synthesis.

As a result of these determinations, the decreased phagocytosis in Fig. 1A (normal RPMI) could have been a reflection of a lower number of macrophages in culture with ADMA for 3 days (Fig. 1D). To make the data easier to interpret, total 4T1 cell consumption by RAW264.7 cells at 10 and 40 min is presented in Fig. 1G and then 4T1 cell consumption is normalized on a per macrophage basis in both the control and ADMA group (Fig. 1H). Under normal RPMI 1640 culture conditions, fewer 4T1 cells were consumed at 10 min by ADMA treated RAW264.7 cells (1.87 ± 0.47 × 10^5^) compared to the control group (3.02 ± 0.54 × 10^5^). However, when normalized to macrophage numbers, tumor cell consumption at 10 min per ADMA treated macrophage was slightly lower but not statistically significantly lower at 0.104 ± 0.026 versus the control group (0.125 ± 0.022). At 40 min, almost all tumor cells were taken (4.83 ± 0.16 × 10^5^ in control group; 4.59 ± 0.10 × 10^5^ in ADMA group), with tumor cell consumption per macrophage at 0.254 ± 0.006 in the ADMA group and 0.200 ± 0.006 in the control. Thus, in normal RPMI, the predominant effect of ADMA is a negative effect on macrophage proliferation.

As mentioned earlier, arginine and lysine in RPMI 1640 normally serve as competitors for ADMA uptake. Therefore, the same analyses were performed using macrophages cultured in arginine- and lysine-free medium. Under such conditions, strikingly fewer 4T1 cells were consumed at 10 min by ADMA treated RAW264.7 cells (0.50 ± 0.18 × 10^5^) compared to the control group (3.36 ± 0.39 × 10^5^). In addition, tumor cell consumption per ADMA treated macrophage was also significantly lower (0.36 ± 0.10) than the control group (0.89 ± 0.12). Again, almost all tumor cells were taken up at 40 min under arginine- and lysine-free conditions (4.53 ± 0.31 × 10^5^ in control group; 4.70 ± 0.18 × 10^5^ in ADMA group). However, at 40 min, tumor cell consumption per macrophage was dramatically higher in the ADMA group (3.45 ± 0.13) versus control (1.20 ± 0.08) since there were about one third the number of macrophages present in the ADMA treated group. Thus, in arginine- and lysine-free RPMI, ADMA markedly reduced macrophage survival and delayed initiation of, but did not eliminate, phagocytosis. In fact, at the later timepoint, ADMA stimulated phagocytosis on a per cell basis.

ADMA is a natural NOS inhibitor [38], but unknown was whether the inhibition of NOS by ADMA in macrophages would favor the use of Arginase (ARG), leading to polarization towards the M2 phenotype. We therefore next determined whether ADMA had any impact on ARG activities. As shown in Fig. 1F, a 2-day ADMA treatment of RAW264.7 cells did not stimulate ARG activity. Taken together, we found ADMA to temporarily, negatively impact the phagocytic capacity of macrophages. ADMA also inhibited the proliferation of macrophages and reduced survival in the absence of arginine and lysine. While ADMA inhibits NOS activity in macrophages, it had no effect on ARG activity.

### ADMA treatment produces an unusual phenotype

**Fig. 2:**
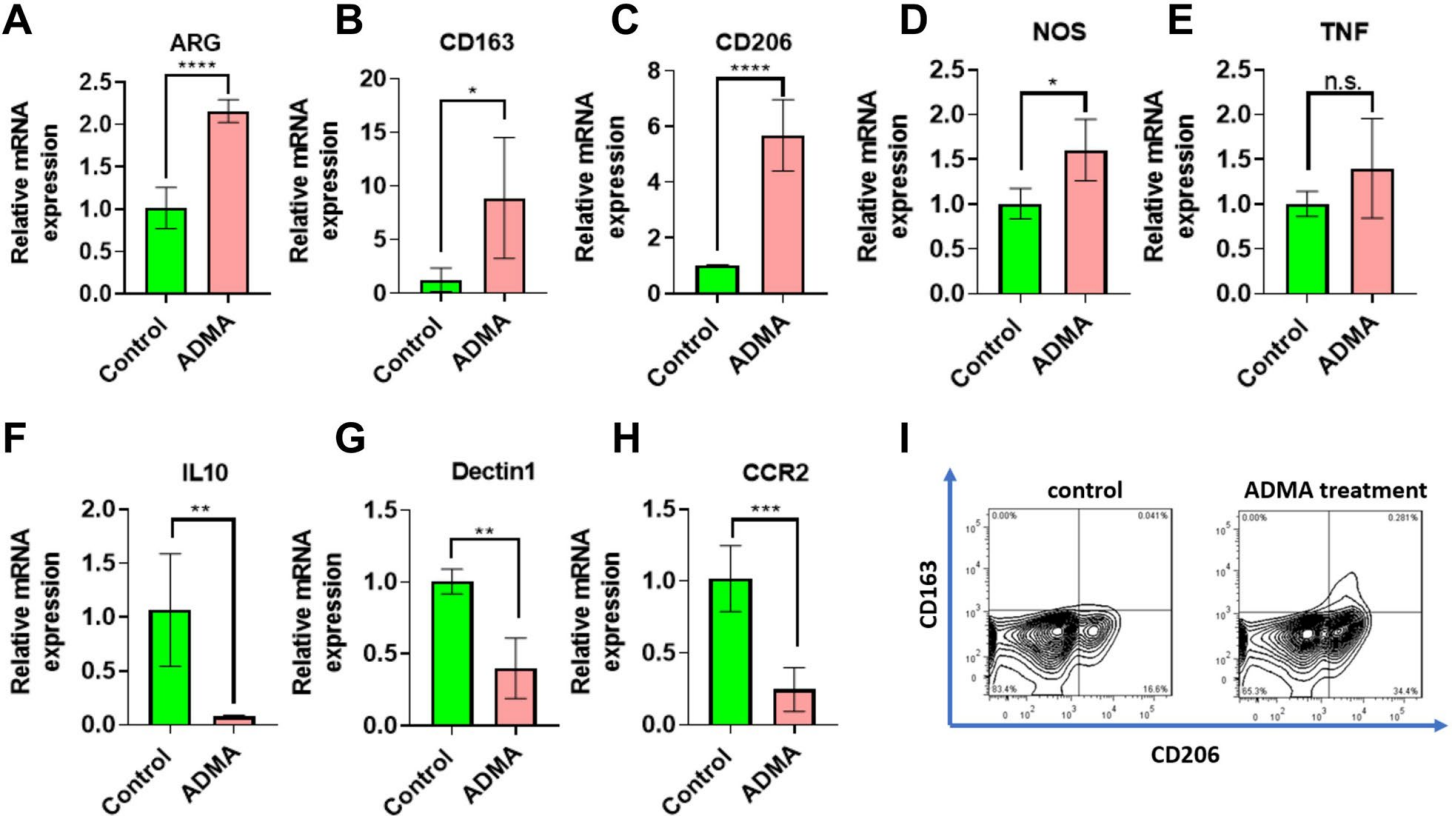
ADMA Drives Macrophages Towards an M2-like subtype. RAW264.7 cells were treat-ed with DPBS (control) or ADMA (5 mM) for 3 days. RNA was collected and expression of M1- and M2-related genes was analyzed. Many M2 related markers were strikingly induced, in-cluding ARG (**A**), CD163 (**B**) and CD206 (**C**) while the M1 related markers NOS (**D**) and TNFα (**E**) were slightly or not impacted by ADMA. However, some other M2 related markers were downregulated such as IL10 (**F**) and Dectin-1 (**G**). Additionally, the chemotactic receptor CCR2 was also downregulated by ADMA (**H**). The most dramatically upregulated M2 markers (CD163 and CD206) by RNA expression were validated at the protein level by flow cytometry (**I**). Data are presented as mean ± SD. (*p < 0.05, *** p<0.001, ****p<0.0001), N=4 for all groups

Even though ARG activity was unaffected by ADMA, we nevertheless examined the expression of genes characteristic of the M1 or M2 phenotypes by qPCR. The expression of several M2-related markers was significantly increased in response to ADMA, including arginase (2.1 fold, Fig. 2A), cd163 (6.8 fold, Fig. 2B) and cd206 (Mannose receptor, 5.7 fold, Fig. 2C) while expression of the monitored M1-related markers was not dramatically altered (nos, 1.6 fold, Fig. 2D and tnf-α,1.4 fold, Fig. 2E). Given the increases in cd163 and cd206, which are associated with the M2c phenotype, it was surprising to see that some M2-related genes were downregulated. Thus, il10 showed a 92% reduction (Fig. 2F) and dectin-1 a 60% reduction (Fig. 2G). ADMA-treated RAW264.7 cells also had reduced expression of ccr2 (76% reduction, Fig. 2H), which in time would be expected to cause reduced chemotaxis of macrophages towards cells producing Ccl2. To determine whether the mRNA expression data were representative of cell surface protein, the expression of the surface CD163 and CD206 which were dramatically upregulated at mRNA level in ADMA- treated RAW264.7 cells was analyzed using flow cytometry. Surface expression of both CD163 and CD206 was increased (Fig. 2I).

Taken together, ADMA treatment appears to produce an unusual (M2-like) phenotype with some impairments such as IL10 production.

### ADMA impacted proliferation but not survival of tumor cells

**Fig. 3:**
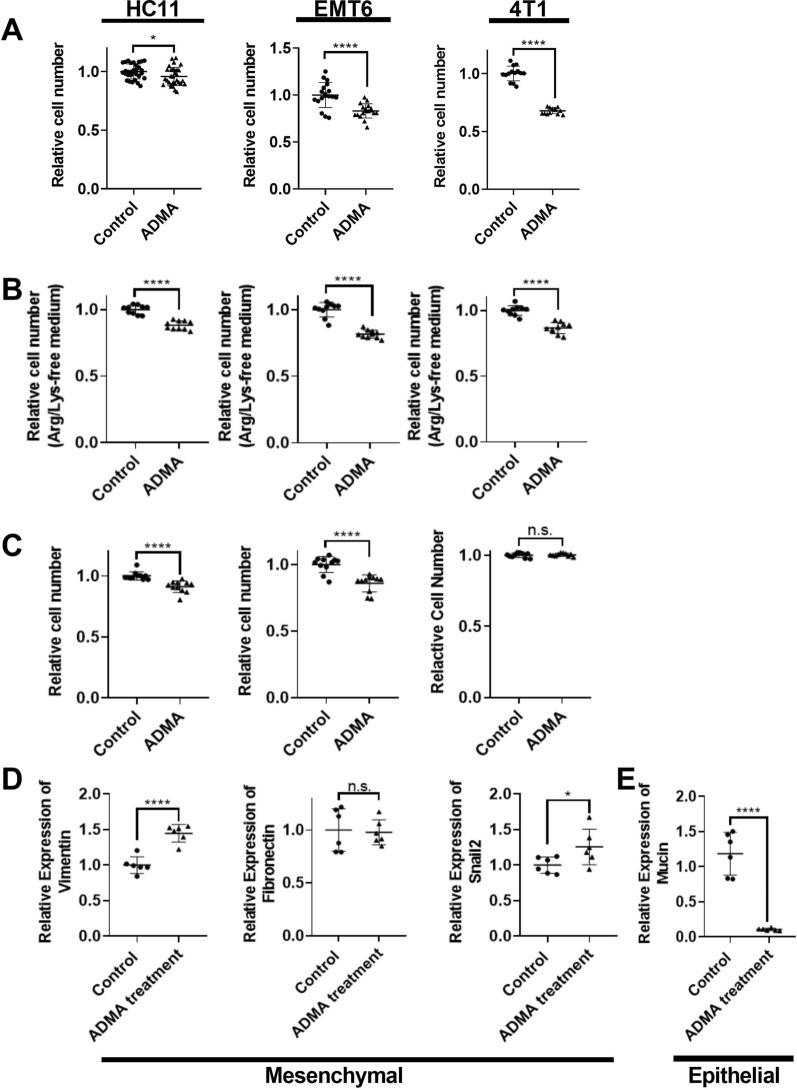
ADMA effect on Tumor Cell Number and Mesenchymal Markers. HC11, EMT6 or 4T1 cells were treated with DPBS (control) or ADMA (5 mM) for 3 days. Cell number was determined by MTS assay. There was a small effect of ADMA on cell growth no matter whether the tumor cells were cultured in regular RPMI 1640 (**A**) or the arginine- and lysine-free RPMI 1640 medium (**B**). Similarly, the same small impact of ADMA on stem cell proliferation was observed (**C**). EMT6 breast cancer cells were used to examine the impact of ADMA on the epithelial to mesenchymal transition (EMT). The mesenchymal related markers, vimentin and snail2, were increased and fibronectin was not affected by ADMA treatment (**D**). Epithelial mucin-1 was decreased by ADMA treatment (**E**). Data are presented as individual replicates with the mean ± SD. (*p < 0.05, *** p<0.001, ****p<0.0001), N=8 (**A**–**C**) and N=6 (**D**,** E**)

Since incubation in ADMA decreased both proliferation (in complete medium) and survival (in arginine- and lysine-free medium) of macrophages, we asked whether it might have similar effects on tumor cells. Like macrophages, ADMA treatment also slowed growth of tumor cells (Fig. 3A). The normal-like HC11 cells seemed less affected (4.3% decreased cell growth by ADMA) than the cancer cell types, EMT6 (16.9% decreased cell growth by ADMA) or the TNBC 4T1 cells (32.2% decreased cell growth by ADMA) (Fig. 3A). In contrast to what was seen in the macrophages, there was a smaller effect on cell growth by ADMA in the cancer cell lines in the arginine- and lysine-free culture medium (18.4% and 13.4% decreased cell growth by ADMA in EMT6 and 4T1 respectively) (Fig. 3B) suggesting that the decrease in growth with ADMA was likely the result of lack of arginine because of the time taken to recycle ADMA back to citrulline for arginine synthesis. Growth of the normal-like HC11 cells was slightly affected by ADMA in the arginine/lysine-free medium (11.9% decreased growth). Similar to the impact on all cells (Fig. 3A and B), ADMA treatment reduced the growth of stem cells (9% and 14.2% decreased growth by ADMA in HC11 and EMT6 respectively) but the malignant 4T1 CSCs seemed to be quite resistant to the ADMA mediated growth impairment (Fig. 3C).

Above, we have suggested that the delayed phagocytic property of macrophages in response to ADMA treatment might contribute to disease process by providing a short window of time for tumor cells to escape from the primary tumor. However, tumor cell escape is dependent on the cells becoming more mobile. We therefore sought to determine if ADMA caused any change in the expression of epithelial or mesenchymal genes. While EMT is regulated by many genes, commonly used mesenchymal markers (vimentin, snail2 and fibronectin) and epithelial markers (mucin, E-cad) were measured to provide some indications in the EMT process. In order to determine potential changes in either direction, we first chose to use EMT6 cells, which among our 3 mouse cell lines sits in the middle of the epithelial to mesenchymal spectrum. Figure 3D shows that among the three mesenchymal genes tested, vimentin (by 44.7%) and Snail2 (by 25.6%) increased. At the same time, the epithelial marker, mucin, was decreased (by 89.5%). Another epithelial marker, E-cadherin was not detectable by qPCR in any circumstance. As an additional test of the effect of ADMA on tumor EMT, we examined EMT using the highly mesenchymal and aggressive 4T1 cell line. As seen in Additional file 1: Fig. S1, exposure of 4T1 cells to ADMA did not further increase the expression of mesenchymal genes (Additional file 1: Fig. S1), while the epithelial gene marker, mucin, was consistently decreased (Additional file 1: Fig. S1B) thus leading to a potential increased mesenchymal status of 4T1 cells. In summary, in contrast to the cytotoxic impact of ADMA on macrophages, ADMA treatment had less impact on proliferation and survival of bulk tumor cells and CSCs. Our results also suggest the possibility that ADMA is involved in the EMT process.

### Exposure to activated immune cells alters the arginine metabolic landscape in cancer stem cell spheroids (CSCsp)

**Fig. 4:**
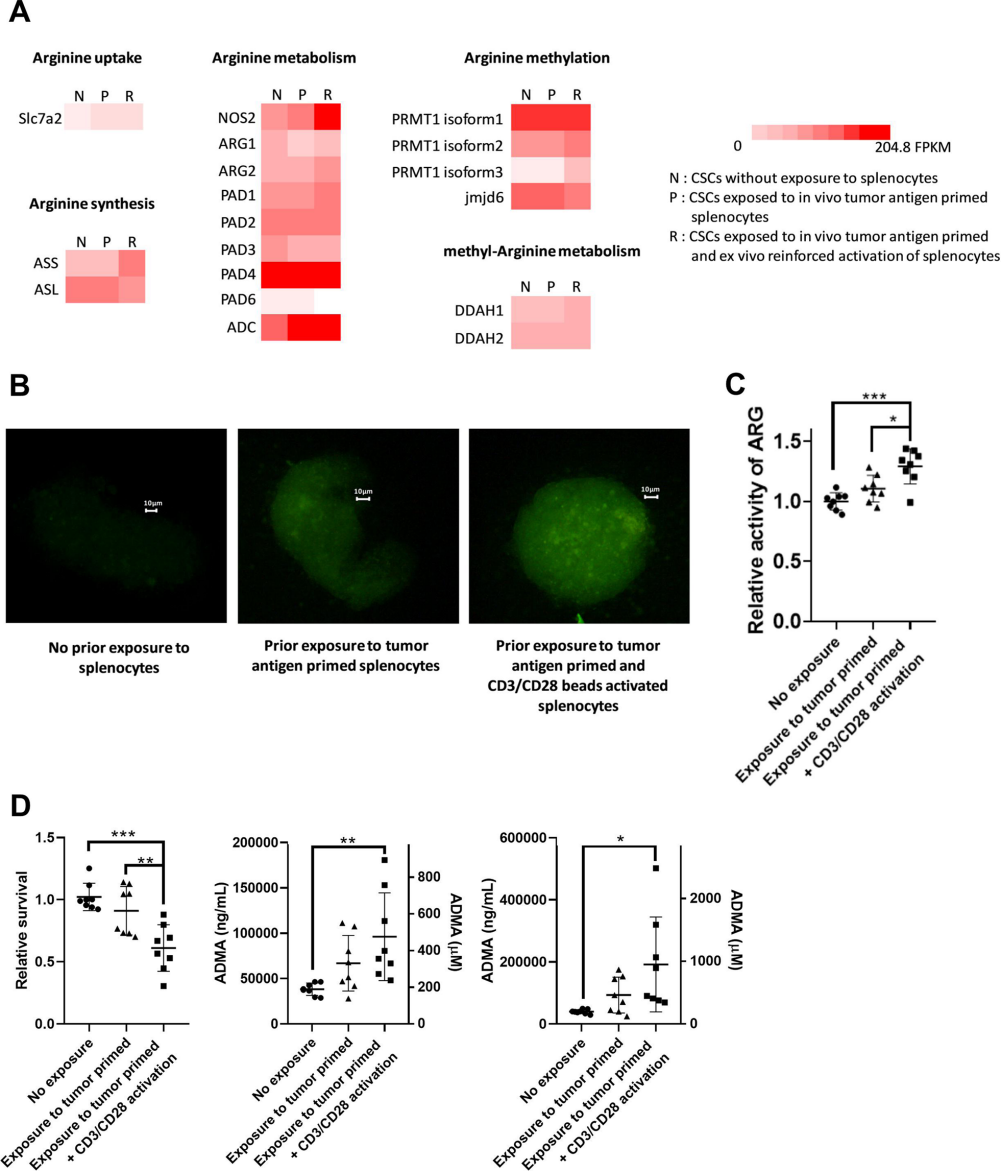
Exposure to Activated Immune Cells Altered the Arginine Metabolic Landscape in Cancer Stem Cells (CSCs). The heatmap compares gene expression involved in Arginine metabolism in 4T1 CSCs that were not exposed to splenocytes (N), exposed to in vivo tumor antigen primed splenocytes (P) or exposed to in vivo tumor antigen primed splenocytes plus reinforced activation by anti-CD3/CD28 dynabeads (R). Significant increases in genes were noted in CSCs exposed to the in vivo tumor antigen-primed splenocytes with reinforced activation by anti-CD3/CD28 dynabeads (**A**). An increase in dansyl-arginine uptake (green fluorescence) was seen in 4T1 CSC spheroids with prior exposure to in vivo tumor antigen primed splenocytes and even more uptake of dansyl-arginine with in vivo tumor antigen primed splenocytes with reinforced by anti-CD3/CD28 dynabeads (**B**). Additionally, the activity of Arginase (C) was also increased in 4T1 CSCs that were exposed to in vivo tumor antigen primed splenocytes with reinforced activation by anti-CD3/CD28 dynabeads. The activity of Arginase was measured through the production of urea (**C**). Immune challenge of 4T1 CSCs by the in vivo tumor antigen primed splenocytes or these with reinforced activation by anti-CD3/CD28 dynabeads decreased tumor cell counts (examined via MTS) and increased secretion of ADMA into the conditioned medium (**D**, left and middle). The ADMA production per 4T1 CSC was plotted (D, right panel). ADMA concentration was expressed as ng/mL (left Y axis) or µM (right Y axis). Data are presented as individual replicates with the mean ± SD. (*p < 0.05, ** p  0.01, ****p < 0.0001), N = 3 for ELISA and N = 4 for ARG enzymatic assay

To investigate if immune challenge of CSCs led to increased ADMA and whether other arginine metabolic pathways were also impacted, 8 week-old Balbc/J female mice were injected with 4T1 tumor cells and housed for 28 days. Splenocytes containing resultant tumor antigen-primed T cells were then collected and a subset had their activation reinforced ex vivo with anti-CD3/CD28 dynabeads. They were then exposed to cultured 4T1 CSCsp for 2 days. The suspended splenocytes were then removed by passage through a cell strainer which retained the 4T1 CSCsp and gene expression in the CSCsp was analyzed by RNAseq. In this experiment, 4T1 CSCsp were cultured alone or with splenic T cells primed in vivo by tumor antigen but with no ex vivo reinforcement of activation, or with the reinforced ex vivo activation of tumor antigen primed splenocytes. Significant changes in the arginine metabolic landscape in CSCsp were observed. These included changes in gene expression likely indicative of an increase in arginine uptake, metabolism and methylation but not in endogenous arginine synthesis (Fig. 4A; Additional file 2: Table S1). Thus, cells acquire environmental arginine through the cationic amino acid transporter 2 [CAT2, also known as solute carrier family 7 (Slc7a2)]. While the expression of Slc7a2 in CSCsp was normally low, incubation with tumor antigen primed splenocytes (whether with reinforced activation or not) increased expression. To translate the increased expression of Slc7a2 into functional outcomes, CSCsp that were cultured alone, or exposed to tumor antigen primed splenocytes or reinforced tumor antigen primed splenocytes were then incubated in arginine/lysine-free medium supplemented with dansyl-arginine. As seen in Fig. 4B, the uptake of dansyl-arginine, which fluoresces green, was increased in CSCsp that were exposed to tumor primed splenocyte preparation but uptake was greater in CSCsp exposed to splenocytes with reinforced activation. The increased uptake of arginine was also consistent with what was observed for downstream arginine metabolism, including increased expression of NOS, Arg2, Protein arginine deiminase (PADI 1, 2 and 4) and arginine decarboxylase (ADC) (Fig. 4A). To confirm the protein activities of these arginine metabolic enzymes, we measured the production of urea in CSCsp as determinants of the activities of Arg2. Urea production increased in CSCsp in response to the presence of splenocytes with reinforced activation (Fig. 4C), again supporting increased function of metabolic enzymes in the arginine pathway.

In addition to measures of arginine uptake and metabolism, it was intriguing to see increased expression of protein arginine methyltransferases (PRMT1, 2 and 3), enzymes responsible for arginine methylation, and decreased expression of jmjd6, an enzyme responsible for arginine demethylation specifically in CSCsp exposed to the reinforced activated splenocytes (Fig. 4A). These results implied potential increased methylation of arginine occurred in CSCsp in response to the presence of activated T cells. ADMA is a metabolite derived from proteolysis of methylated proteins. To determine whether exposure to the splenocytes with reinforced activation resulted in higher ADMA generation, we examined ADMA in the conditioned medium. More ADMA was detected in conditioned medium from CSCsp exposed to tumor primed and reinforced splenocytes when compared to CSCsp cultured alone or with just tumor primed splenocytes (Fig. 4D, middle panel). Since these tumor-primed and reinforced activated splenocytes could potentially lead to immune attack of CSCsp, we assessed relative tumor cell number by MTS assay. Fewer cells were present in CSCsp when incubated with the tumor primed and reinforced activated splenocytes (Fig. 4D, left panel). As ADMA generation has to occur in live cells (due to the requirement for active proteasomal or autophagosomal degradation and transport of ADMA via the ATP-dependent transporter, Slc7a2), ADMA in the medium was not due to apoptosis (induced by activated splenic T cells) or rupture of tumor cells. The presence of ADMA in the environment was therefore likely contributed solely by the remaining live CSCsp. The amount of ADMA produced by live CSCsp was therefore calculated by dividing the amount of ADMA in the conditioned medium relative to the CSCsp population and this calculation is presented in Fig. 4D, right panel. Taken together, these data support the contention that CSCsp modulate their arginine metabolic landscape in response to immune attack, including increased uptake of arginine, increased metabolism of arginine, and increased methylation of arginine in proteins.

### Interferon γ-mediated autophagy contributes to the production of ADMA

**Fig. 5:**
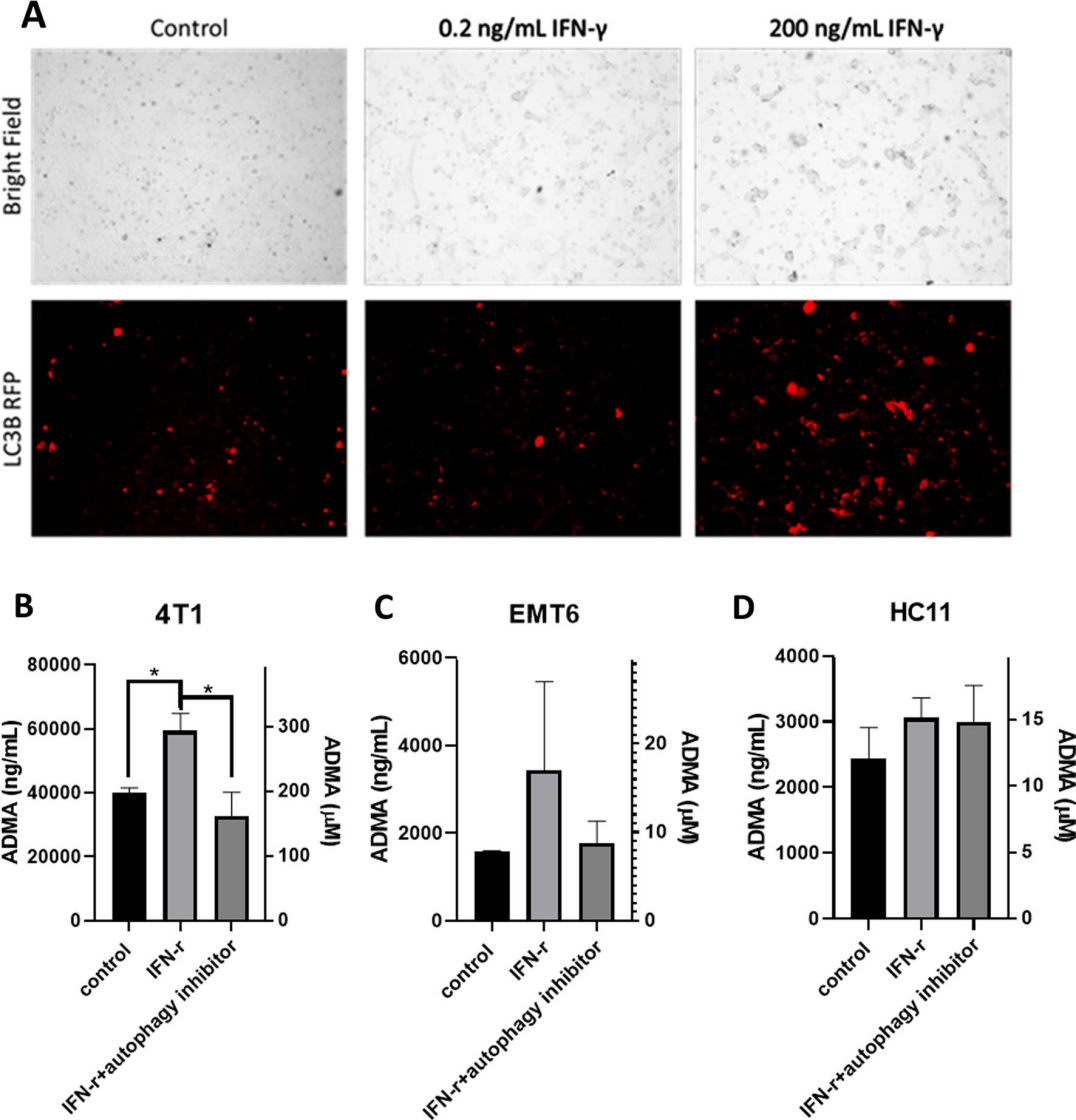
Interferon γ-mediated autophagy contributes to the production of ADMA. 4T1 breast cancer cells were treated with low dose (0.2 ng/mL) or high dose (200 ng/mL) IFN-γ for 3 days. Induction of autophagy was measured using Premo LC3B-RFP viral transduction. The high dose of IFN-γ induced autophagy of 4T1 cells (**A**). The same dose of IFN-γ also led to increased secretion of ADMA into the conditioned medium in mouse breast cancer 4T1 cells (**B**) and IFN-γ induced ADMA secretion was blocked in 4T1 cells when 250 nM autophagy inhibitor was added to the culture (**B**). The effect of IFN-γ on the EMT 6 (**C**) or mouse normal mammary epithelial cell line, HC11, was not significant and unaffected by the autophagy inhibitor (**D**). ADMA concentration was expressed as ng/mL (left Y axis) or µM (right Y axis). Data are presented as mean ± SEM. (*p < 0.05, ***p < 0.001, ****p < 0.0001), N = 5

As arginine cannot be directly methylated as an amino acid monomer, methylation of arginine occurs in proteins. ADMA is therefore generated from protein degradation either through proteasomal degradation or autophagy. Above, we have shown that tumor cells increased arginine methylation and ADMA production in response to activated spleen cells. One of the immune strategies to fight cancers is via the secretion of cytokines. Because IFN-γ has been reported to induce autophagy in hepatocellular tumor cells [39], we hypothesized that IFN-γ might induce autophagy in breast cancer cells and that autophagic breakdown of cellular proteins could be a major source of ADMA. To test this hypothesis, we treated 4T1 cells with a low concentration (0.2 ng/mL) of IFN-γ, known not to induce autophagy in liver cancer cells, as a negative control and a high dose of IFN-γ (200 ng/mL), proven to induce autophagy in liver cancer cells, and examined the expression of the autophagy marker, LC3B. As seen in Fig. 5A, the high dose of IFN-γ treatment increased the number of red fluorescing LC3B^+^ cells, indicative of increased autophagy. We next aimed to determine if the autophagy induced by IFN-γ contributed to the generation and secretion of ADMA by tumor cells.

In these experiments, total bulk tumor cells and not CSCsp were used. Mouse breast cancer EMT6 and 4T1 cells were treated with the high dose of IFN-γ (200 ng/mL), the diluent as a control, or the high dose of IFN-γ in the presence of an autophagy inhibitor for 3 days. Conditioned medium was then collected and the ADMA in the conditioned medium was measured using an ELISA. As shown in Fig. 5B–D, treatment with IFN-γ stimulated the generation and secretion of ADMA by 4T1 tumor cells and inhibition of this by the inhibitor, thereby demonstrating an important contribution to ADMA production through autophagy. The effect of IFN- γ on ADMA generation was not significant in EMT6 cells and the normal epithelial HC11 cells, either in the absence or presence of the autophagy inhibitor (Fig. 5C, D). Some autophagy inhibitors have been shown to have cytotoxicity. To ensure that the results showing decreased ADMA production by tumor cells was not an outcome of decreased cell number, we also examined the cellular viability of HC11, EMT6 and 4T1 cells in response to the same concentration (250 nM) and duration (3 days) of autophagy inhibitor treatment and no difference was observed in cellular viability across all three cell lines (Additional file 1: Fig. S2A–C). To further validate that ADMA production was not affected by the autophagy inhibitor even in the absence of an effect on viability, an ELISA was conducted to examine secreted ADMA from conditioned medium of EMT6 and 4T1 cells treated with the autophagy inhibitor. Again, no impact on ADMA secretion was seen in response to the autophagy inhibitor (Additional file 1: Fig. S2D).

### ADMA is produced by both bulk tumor and cancer stem cells

**Fig. 6:**
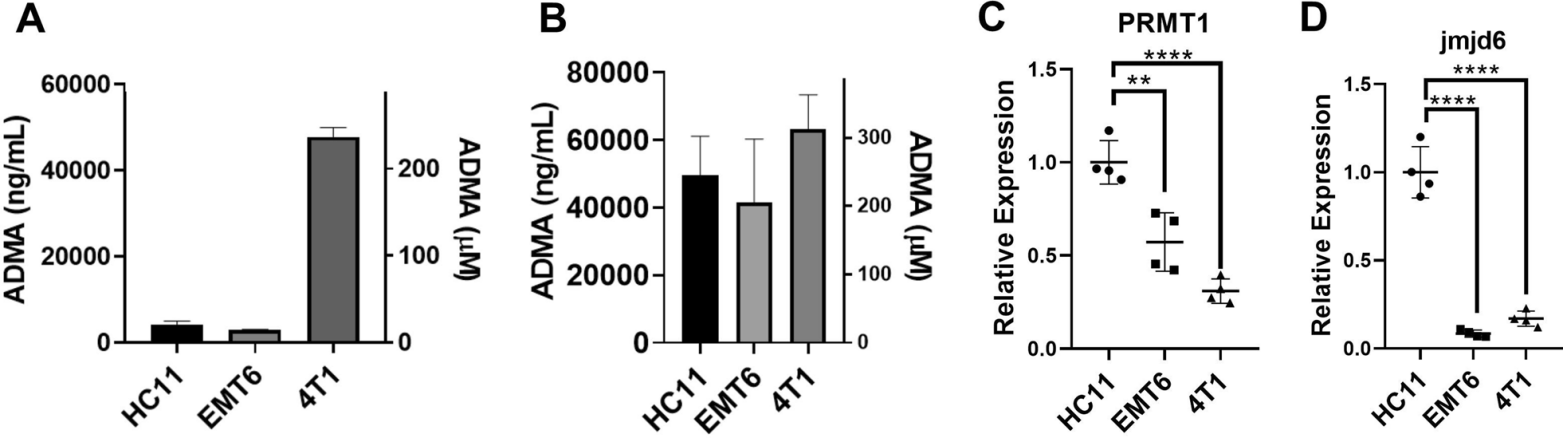
All Normal, Bulk Tumor Cells and CSCs Made ADMA but the Expression of PRMT1 and Jmjd6 was Different. All cell lines including mouse normal-like epithelial HC11, mouse breast cancer EMT6 and 4T1 cells were capable of generating and secreting ADMA into conditioned medium (**A**). The same was also observed using the stem cell cultures (**B**). RNA expression analyses showed that normal- like HC11 cells had high expression of the arginine methylating enzyme, PRMT1 (**C**) but also very high expression of the arginine demethylating enzyme, jmjd6 (**D**). The tumor cells, EMT6 and 4T1, by contrast, expressed lower levels of PRMT1 (C) and even lower relative levels of jmjd6 (**D**). ADMA concentration was expressed as ng/mL (left Y axis) or µM (right Y axis). Data are presented as individual replicates or mean ± SD. (*p < 0.05, ** p < 0.01, ****p < 0.0001), N = 5 for all ELISA (**A**, **B**, **E**) and N = 4 for the real-time PCR analyses (**C**, **D**)

To investigate if bulk tumor cells or cancer stem cells (CSCs) could contribute to the elevated serum ADMA in tumor patients, we sought to determine the production and release of ADMA by bulk tumor cells or CSCs. Conditioned medium was collected from the bulk tumor or CSC cultures and the concentration of ADMA in the conditioned medium was quantified by sandwich ELISA. All cell lines, including the normal-like breast epithelial HC11 cells, the breast cancer EMT6 cells, and the TNBC 4T1 cells (Fig. 6A) and their equivalent stem cell culture (Fig. 6B) secreted ADMA. The secreted ADMA was normalized to the relative cell numbers at the time of sample collection. The bulk 4T1 cells secreted much more ADMA than the other two lines, a difference that was eliminated when examining only CSCs. While our results also suggested that CSCs seemed to generate more ADMA than bulk tumor cells with the exception of 4T1 cells, we avoided such overinterpretation as the culture medium and plate (ultralow attachment plate for the stem cell culture) might have affected other gene expression and cellular activities. As ADMA is generated from methylated arginine in proteins, we therefore examined the expression of two key enzymes, PRMT1 (protein arginine methyl transferase 1) that is responsible for arginine methylation and jmjd6 (jumonji domain containing protein 6) which is responsible for arginine demethylation in all cell lines tested. PRMT1 expression was lower in both EMT6 and 4T1 tumor cells compared to the normal-like HC11 cells (Fig. 6C). However, both tumor cell lines (EMT6 and 4T1) expressed extremely low jmjd6 (Fig. 6D). Thus, these results imply there might be some potential dysregulation of protein arginine methylation and demethylation in tumor cells.

Thus, data from Figs. 5 and 6 show that all tested cells are capable of secreting ADMA and that interferon-γ exposure, as would occur with immune attack, increases the production of ADMA in tumor cells.

## Discussion

Elevated plasma ADMA is found in a variety of human diseases including cardiovascular disease [26] and cancer [27–30]. Because of the longer history in cardiovascular disease and the inhibitory effect of ADMA on NOS (nitric oxide synthase), analysis of ADMA in cancer has thus far focused on endothelial-related functions such as tumor angiogenesis and metastasis [31, 32]. However, the impact of elevated serum ADMA on tumor-immune interactions is not known. In the current study, we have addressed the impact of ADMA on both macrophages and tumor cells and their interactions.

For macrophages, the impact would depend on the balance between ADMA and arginine and lysine in the tumor microenvironment. With relatively high ratios of arginine and lysine to ADMA, as would be the case in normal RPMI 1640 and in vitro co-culture, the greatest effect of local ADMA was on macrophage proliferation. With lower ratios, as would occur in less vascular solid tumors and demonstrated herein by culture in arginine- and lysine-free RPMI 1640, there was both decreased macrophage survival and a delay in the onset of phagocytosis of tumor cells. This delay was transient since at 40 min of phagocytosis, the remaining macrophages had surpassed the controls on a per cell basis. Thus, we can conclude that ADMA does not affect phagocytosis per se but only the number of macrophages and some initiating event for phagocytosis. As part of the innate immune system, macrophages rely on the expression and activation of surface pattern recognition receptors (PRR) to initiate function. The C-type lectin receptor, dectin-1, on the surface of macrophages assists in the recognition of tumor cells, which tend to overexpress surface N-glycan structures [40]. Since we found ADMA to decrease dectin-1 expression, reduced dectin-1 may account for the slower phagocytosis initiation in response to ADMA.

Arginine catabolism is required for macrophage activation and proliferation [41]. Arginine, lysine and ADMA share the same ATP-dependent transporter, Slc7a2 (also known as CAT2), required to enter cells [41, 42]. The presence of elevated ADMA in the culture would therefore interfere with the immediate availability of arginine and lysine for macrophages. Reduced proliferation is therefore likely a result of reduced arginine (and lysine) uptake.

Macrophage death is increased by ADMA in arginine- and lysine-free medium. While ADMA can be converted into citrulline for arginine synthesis, it is likely that this is not sufficient to maintain the biosynthetic needs of macrophages. Although the depletion of arginine and lysine in culture medium is an artificial situation, we provide evidence herein that this is likely to happen when CSCs are threatened by immune cells. Under such conditions, the CSCs increased their arginine uptake, metabolism and methylation. This would create a microenvironment around the cancer cells in which arginine was depleted. Not only would this affect macrophage viability but also effector T cells [43–47].

Macrophage polarization is an important process through which macrophages take on distinct functions in response to different environmental stimuli. Macrophage polarization forms a spectrum, with M1 and M2 polarization at opposite extremes. Most macrophages display a phenotype in between the two extremes and, after an initial period within tumors, they are more M2-like. For example, tumor associated macrophages can be classified as M2d macrophages. M2d macrophages express many M2 related markers but also have significant expression of iNOS, an M1 marker [48, 49]. Among many macrophage polarization markers, arginine is also the central player for control of macrophage polarization [16]. Our results demonstrate that ADMA treatment increases important M2 phenotype markers, such as arginase, CD163 and CD206, although the activity of arginase does not increase in the time frame analyzed. At the same time, expression of other M2 markers, IL10 and dectin-1, was decreased. While IL10 is an M2 marker, others have found there was no detectable IL10 expression in M2 macrophages under conditions of arginine deprivation [50]. Confusing the picture is the fact that IL10 secretion by M2 macrophages is crucial for their immune suppressive function. In addition, insufficient IL10 production by M2 macrophages impairs vasculogenesis by endothelial cells [51]. However, because arginine is required for effector T cell function and macrophage activation, it is possible that IL10 may play a minor role when there is low or no arginine in the environment since there is no or low immune activation.

Our results also showed decreased expression of Ccr2 in macrophages exposed to ADMA. Ccr2 is a chemoreceptor necessary for monocytes/macrophages to migrate towards Ccl2, a chemokine produced by a variety of cells, including tumor cells. Thus, our study provides the first indication of the regulatory role of ADMA on macrophage chemoattraction. Future investigation is required to explore macrophage mobility in vivo in response to tumor derived Ccl2 and ADMA.

Since in vivo, ADMA accumulation would likely reduce recruitment of macrophages, delay initiation of macrophage phagocytosis and produce a more M2-like phenotype, we hypothesize that this would provide time for some primary tumor cells to metastasize. Epithelial to mesenchymal transition is a key driver for tumor metastasis and is a dynamic process co-opted by cancer cells during invasion. There is currently no quantification method to define the absolute epithelial or mesenchymal status of cancer cells [52], and cancer cells can display hybrid states ranging from being fully mesenchymal or fully epithelial [52–54]. To investigate if ADMA treatment has any impact on tumor epithelial to mesenchymal transition, we first chose to analyze expression of several commonly examined EMT genes in this cell line (EMT6) which sits between normal-like epithelial HC11 cells and the highly metastatic and more mesenchymal-like 4T1 cells because of the potential to see changes in both mesenchymal and epithelial genes. ADMA increased expression of two mesenchymal, and decreased expression of an epithelial, gene in the EMT6 cells suggesting an impact favoring “mesenchymal like” transition. A similar reduction in the same epithelial gene by ADMA was observed in 4T1 cells, however, no change in the examined mesenchymal genes was noted in the 4T1 cells, likely due to their already highly mesenchymal nature. Thus, with increased ADMA one would predict both reduced immune attack on and greater metastatic potential of tumor cells.

While ADMA had a significant impact on macrophage proliferation and survival, we show proliferation of bulk tumor cells and CSCs were less affected by ADMA regardless of whether arginine was depleted from the culture medium or not. ADMA is normally metabolized into citrulline by the enzyme, DDAH I (dimethylarginine dimethylaminohydrolase I) [22]. The citrulline can then be used for the biogenesis of arginine. Analyses from the TCGA database show expression of DDAH I is significantly increased in almost all cancer types with the exception of lung, melanoma, renal and testicular germ cell tumors [55], although in melanoma the protein is upregulated [56]. Thus, the elevation in DDAH I expression we observed in 4T1 CSCs in response to activated splenocytes potentially contributes to increased ADMA metabolism and therefore arginine biogenesis. This may be why the tumor cells were not as affected by ADMA exposure in regular or arginine/lysine-free medium.

All human cells can make and secrete ADMA [55]. Our results using the mouse normal-like HC11 cells, and two tumor cells (EMT6, and TNBC 4T1) agree with this. However, what causes the elevation of plasma ADMA in cancer patients (1.5–2.5 fold normal value) is unknown. Here, we show that CSCs could be a potential source of ADMA in cancer patients. Upon stimulation by immune surveillance, CSCs increase ADMA secretion, resulting in ~ 200 µg/mL (~ 1 mM) ADMA in mixed whole culture medium. This suggests CSCs as important contributors to the elevation of ADMA in cancers, with a gradient starting high at the CSCs and decreasing with dilution into body fluids. While bulk cancer cells do not produce as much ADMA as CSCs, we show that interferon-γ (IFN- γ), which is produced by activated macrophages and T effector cells, causes 4T1 bulk tumor cells to increase ADMA secretion. The effect is not large and so IFN-γ may not be the only stimulus unless IFN-γ is sufficiently elevated in the circulation and has this effect on many cell types throughout the body. Many factors may contribute to these differences in ADMA production but a likely cause is relative sensitivity to IFN- γ (such as lower expression of IFN- γ receptor). Both the relative expression of IFN- γ receptor and sensitivity to IFN- γ across different tumor cell lines should be further investigated. IFN- γ driven ADMA production is not significant in either EMT6 cancer cells or the normal mammary epithelial HC11 cells, implying that IFN- γ induced ADMA generation might be associated with the malignant capacity of cells. Jmjd6 is an arginine demethylase which preferentially demethylates asymmetric demethylation thus potentially lowering ADMA generation [57–59]. However, the impact of jmjd6 expression on tumor progression is contradictory and dependent on the tumor type [60–62]. The relative expression level of PRMT and jmjd6 therefore may provide additional insights for the regulation of ADMA production in tumor cells, depending on specific tumor type.

ADMA cannot be made by direct methylation of free mono-arginine but is instead made via proteolysis (either through autophagosome or proteasome). Proteasome inhibitors block the generation of both ADMA and SDMA (symmetric dimethylarginine) and autophagy inhibitors only block the production of ADMA [63]. Therefore, proteolysis via proteasomes can lead to the generation of two methylated arginine products, ADMA and SDMA, while autophagy only leads to the generation of ADMA. Effector T cells exert their cytotoxic function via lytic pathways (release of death ligand, perforin or granzyme B) plus non-lytic pathways (through release of cytokines such as IFN- γ). IFN- γ is a known autophagy inducer [39, 64]. Consistent with this, we show that IFN- γ treatment increases the ADMA in the conditioned medium by tumor cells and IFN- γ treatment also induces autophagy of 4T1 bulk tumor cells. In addition, an autophagy inhibitor blocks IFN- γ-driven ADMA generation. In addition to tumor cell autophagy, arginine deprivation induces T cell autophagy [65]. The increased consumption of arginine by tumor cells could potentially deprive environmental arginine and promote T cell autophagy, which may add to local ADMA as well as inhibit T effector cell anti-tumor activities. Future studies will focus on ADMA and T effector cell anti-tumor function.

## Conclusion

In conclusion, this work provides a mechanistic understanding of a novel role for ADMA in tumor progression through its multiple effects on macrophages. Extracellular ADMA affects macrophage phenotype, slows phagocytosis of tumor cells, likely reduces recruitment of monocytes and reduces proliferation and survival of macrophages, thereby creating an immune escape for tumor cells. Due to overexpression of DDAH I in most tumor types, ADMA has only a marginal impact on proliferation and survival of tumor cells themselves but does encourage the epithelial to mesenchymal transition of tumor cells necessary for effective metastasis. Autophagy, driven by IFN-γ, is a possible cause for the elevation of plasma ADMA in cancer patients. Understanding the role of ADMA allows for the development of novel anti-cancer therapeutics which may also be relevant in cardiovascular disease.

## Supplementary Information

**Additional file 1: Figure S1.** ADMA had Minor Impacts on the Epithelial to MesenchymalTransition of 4T1 Tumor Cells. 4T1 breast cancer cells were used to examine theimpact of ADMA on the epithelial to mesenchymal transition (EMT). None of theexamined mesenchymal genes were affected by ADMA treatment (A).  The epithelial mucin-1 was decreased by ADMAtreatment (B). Data are presented as individual replicates with the mean ± SD.( ** p < 0.01). **Figure S2.** Neither Cellular Viability nor ADMA Production was Impacted by Autophagy Inhibitor. Autophagy inhibitor (250 nM) was used to treat 3 mouse breastcell lines for 3 days. At day 3, cell viability was measured by MTS assay andno observed cytotoxicity was seen in all 3 cell lines (A). At day 3,conditioned medium was collected from EMT6 and 4T1 cells to further analyze theADMA generation and secretion under autophagy inhibitor stimulation. Similarly,no significance was found (B) Data are presented as individual replicates withthe mean ± SD.

**Additional file 2: Table S1**. RNA sequencing depicts alteration of arginine metabolism in 4T1 CSCs exposed to activated immune cells (FPKM). **Table S2.** Primer sequences used for qPCR analyses.

## Data Availability

The dataset supporting the conclusions of this article is available from the corresponding author. RNAseq data are available in the GEO database at NCBI with the accession number GSE199983 (https://www.ncbi.nlm.nih.gov/geo/query/acc.cgi?acc=GSE199983).

## Abbreviations

ADMA: Asymmetric dimethylarginine
ARG: Arginase
CSC: Cancer stem cells
CSCsp: Cancer stem cell spheroids
DDAH: Dimethylarginine dimethylaminohydrolase
IFN- γ: Interferon gamma
NOS: Nitric oxide synthase
