# Supplementary material for: Tumor cell-derived asymmetric dimethylarginine regulates macrophage functions and polarization
Source: Cancer Cell Int. 2022 Nov 15;22:351. doi: 10.1186/s12935-022-02769-7 (PMC9664648; doi:10.1186/s12935-022-02769-7)
Supplement: Supplementary file 1 — Additional file 1: Figure S1. ADMA had Minor Impacts on the Epithelial to MesenchymalTransition of 4T1 Tumor Cells. 4T1 breast cancer cells were used to examine theimpact of ADMA on the epithelial to mesenchymal transition (EMT). None of theexamined mesenchymal genes were affected by ADMA treatment (A). The epithelial mucin-1 was decreased by ADMAtreatment (B). Data are presented as individual replicates with the mean ± SD.( ** p < 0.01). Figure S2. Neither Cellular Viability nor ADMA Production was Impacted by Autophagy Inhibitor. Autophagy inhibitor (250 nM) was used to treat 3 mouse breastcell lines for 3 days. At day 3, cell viability was measured by MTS assay andno observed cytotoxicity was seen in all 3 cell lines (A). At day 3,conditioned medium was collected from EMT6 and 4T1 cells to further analyze theADMA generation and secretion under autophagy inhibitor stimulation. Similarly,no significance was found (B) Data are presented as individual replicates withthe mean ± SD. [file 12935_2022_2769_MOESM1_ESM.pdf]

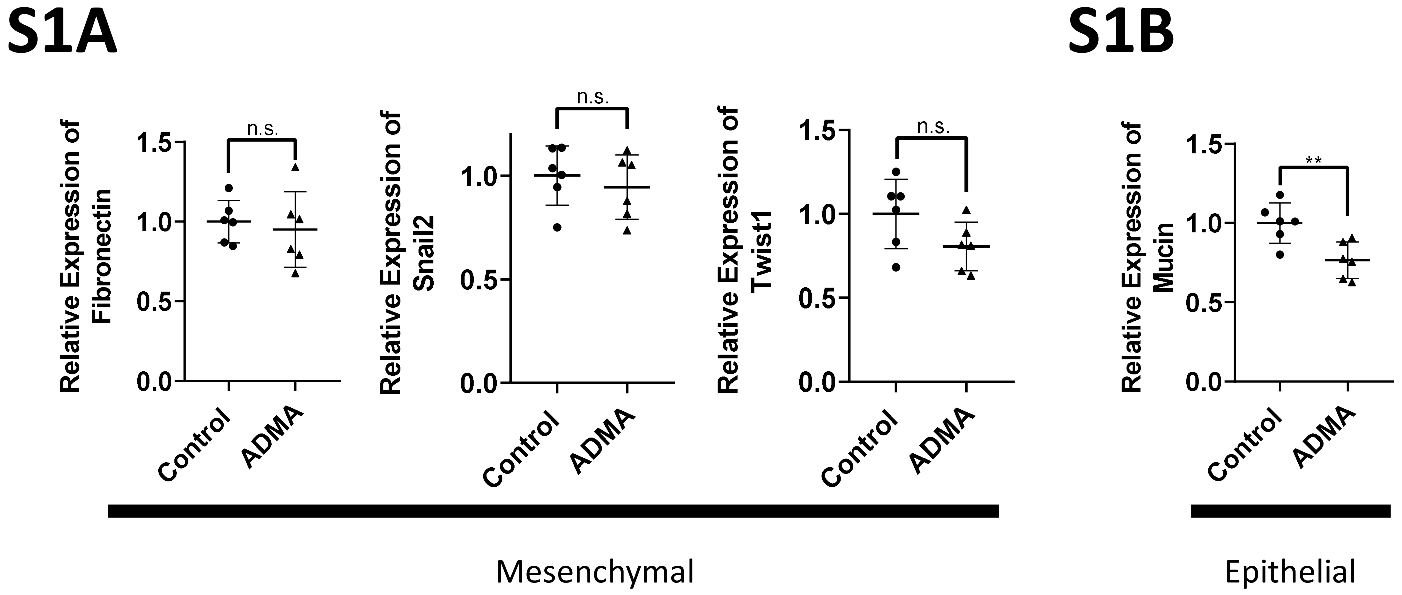

Supplementarty Figure 1. ADMA had Minor Impacts on the Epithelial to Mesenchymal Transition of 4T1 Tumor Cells. 4T1 breast cancer cells were used to examine the impact of ADMA on the epithelial to mesenchymal transition (EMT). None of the examined mesenchymal genes were affected by ADMA treatment (A). The epithelial mucin-1 was decreased by ADMA treatment (B). Data are presented as individual replicates with the mean ± SD. (** p<0.01)

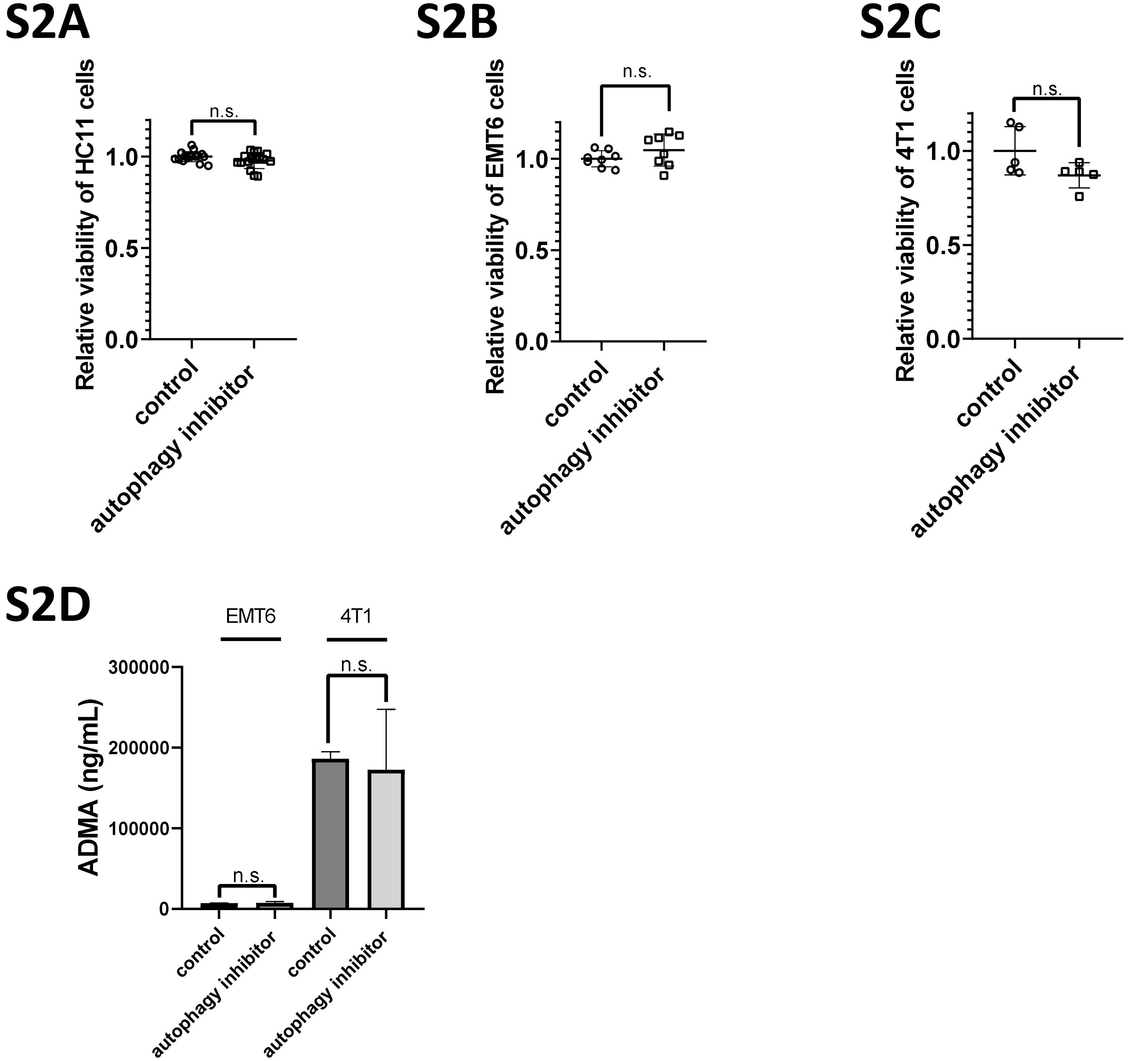

Supplementarty Figure 2. Neither Cellular Viability nor ADMA Production was Impacted by Autophagy Inhibitor. Autophagy inhibitor (250 nM) was used to treat 3 mouse breast cell lines for 3 days. At day 3, cell viability was measured by MTS assay and no observed cytotoxicity was seen in all 3 cell lines (A). At day 3, conditioned medium was collected from EMT6 and 4T1 cells to further analyze the ADMA generation and secretion under autophagy inhibitor stimulation. Similarly, no significance was found (B) Data are presented as individual replicates with the mean ± SD.
